# A Murine CD8^+^ T Cell Epitope Identified in the Receptor-Binding Domain of the SARS-CoV-2 Spike Protein

**DOI:** 10.3390/vaccines9060641

**Published:** 2021-06-11

**Authors:** Jihyun Yang, Eunjin Kim, Jong-Soo Lee, Haryoung Poo

**Affiliations:** 1 Infectious Disease Research Center, Korea Research Institute of Bioscience and Biotechnology (KRIBB), Daejeon 34141, Korea; jhyang@kribb.re.kr (J.Y.); ejkim1512@gmail.com (E.K.); 2 Department of Preventive Veterinary Medicine, College of Veterinary Medicine, Chungnam National University, Daejeon 34134, Korea; jongsool@cnu.ac.kr

**Keywords:** SARS-CoV-2, cell-mediated immunity, CD8 cytotoxic T lymphocyte, epitope, vaccine

## Abstract

The ongoing COVID-19 pandemic caused by SARS-CoV-2 has posed a devastating threat worldwide. The receptor-binding domain (RBD) of the spike protein is one of the most important antigens for SARS-CoV-2 vaccines, while the analysis of CD8 cytotoxic T lymphocyte activity in preclinical studies using mouse models is critical for evaluating vaccine efficacy. Here, we immunized C57BL/6 wild-type mice and transgenic mice expressing human angiotensin-converting enzyme 2 (ACE2) with the SARS-CoV-2 RBD protein to evaluate the IFN-γ-producing T cells in the splenocytes of the immunized mice using an overlapping peptide pool by an enzyme-linked immunospot assay and flow cytometry. We identified SARS-CoV-2 S_395–404_ as a major histocompatibility complex (MHC) class I-restricted epitope for the RBD-specific CD8 T cell responses in C57BL/6 mice.

## 1. Introduction

Severe acute respiratory syndrome coronavirus 2 (SARS-CoV-2) is the causative pathogen of human coronavirus disease 2019 (COVID-19) [1,2]. COVID-19 was declared as a pandemic by the WHO in 2020 and has inflicted tremendous negative impacts on global health, society, and economic institutions. As vaccination is considered to be one of the most effective methods for preventing the rapid global spread of this disease, many researchers and companies in the world are attempting to develop safe and effective COVID-19 vaccines [3]. Most COVID-19 vaccine candidates have focused on the SARS-CoV-2 spike protein or the receptor-binding domain (RBD) of the spike protein as the vaccine antigen [4]. The SARS-CoV-2 RBD primarily binds to angiotensin-converting enzyme 2 (ACE2) and facilitates the entry of the virus into host cells [5]. The protective efficacy of the vaccine is conferred by the induction of humoral immunity such as neutralizing antibodies and cell-mediated immunity. CD8 cytotoxic T lymphocytes (CTLs) play a critical role in eliminating virus-infected cells, thereby leading to the effective clearance of viral infection. In particular, CTL activity is important to provide long-term protection from reinfection of the virus [6]. Recently, a clinical report also showed the importance of T cell immunity in COVID-19 recovery [7]. Of patients with severe COVID-19, a significantly higher expression of surface markers for T cell activation was observed in the recovering group than the severe group, whereas no significant differences were observed for neutralizing antibodies in both groups. To analyze the CTL activity induced by SARS-CoV-2 vaccines, the overlapping peptide pool for T cell stimulation was used due to the CTL epitopes of SARS-CoV-2 presently being unidentified [8,9,10]. A recent study reported SARS-CoV-2 S_526–533_ as a CTL epitope in BALB/c mice, through the experimental evaluation of programming-predicted peptides [11]. C57BL/6 mice have been widely used for the evaluation of SARS-CoV-2 vaccines [8,9]. Transgenic mice with a C57BL/6 genetic background expressing human ACE2 (hACE2) are currently being used as an animal model of SARS-CoV-2 study [12,13,14,15]; however, the CTL epitope in C57BL/6 mice has not yet been identified. Additionally, many experimental approaches aimed at quantifying SARS-CoV-2-specific CTL activity have used RBD peptide pools, which are associated with limitations such as high cost and time-consuming synthesis of peptides [8,10]. Thus, finding a murine CTL epitope is important to resolve the limitations and to identify a peptide that can be used for exactly analyzing SARS-CoV-2-specific CD8 T cell activity when evaluating SARS-CoV-2 vaccine candidates in an animal model. In this study, we identified a murine CD8 T cell epitope of the SARS-CoV-2 spike RBD using C57BL/6 wild-type and hACE2-expressing transgenic (K18-hACE2) mice. To our knowledge, this is the first report to indicate that SARS-CoV-2 S_395–404_ is a CD8 T cell epitope in the RBD of the SARS-CoV-2 spike protein in a C57BL/6 mouse model.

## 2. Materials and Methods

### 2.1. Mice

C57BL/6 wild-type (OrientBio, Gyeonggi-do, Korea) and K18-hACE2 transgenic mice (Jackson Laboratory) were housed in a specific pathogen-free facility at the Korea Research Institute of Bioscience and Biotechnology (KRIBB). Mouse care and experiments were reviewed and conducted under guidelines of the Institutional Animal Care and Use Committee of KRIBB (KRIBB-AEC-20279).

### 2.2. Reagents

Recombinant SARS-CoV-2 spike RBD protein was purchased from Sino Biologics (Shanghai, China) and produced in HEK293 mammalian cells using a codon-optimized plasmid encoding SARS-CoV-2 RBD (amino acid residues (aa) 319–541 of SARS-CoV-2 S (accession no. YP_009724390.1)). The Sigma Adjuvant System (SAS), composed of monophosphoryl lipid A and squalene, was obtained from Sigma–Aldrich. Synthetic RBD peptides (8–11-mer and 15-mer peptides) were chemically synthesized by AnyGen (Gwangju, South Korea) and Peptron (Daejeon, South Korea) (summarized in Table 1 and Table 2). The endotoxin-free EndoFit OVA protein and OVA_257–264_ peptide were obtained from InvivoGen (San Diego, CA, USA) and AnaSpec (San Jose, CA, USA), respectively. Mouse IgG2a isotype control monoclonal antibody (mAb) was obtained from Bio X Cell (West Lebanon, NH, USA), and fluorescent dye-conjugated antibodies, anti-H2-K^b^ and anti-H2-D^b^ mAbs, were obtained from BD Biosciences (San Jose, CA, USA).

### 2.3. Immunization

Six- to 10-week-old female mice were randomly distributed in groups of 3 to 5 mice and intramuscularly (i.m.) immunized with 5 μg of RBD recombinant protein combined with SAS on days 0 and 14. In a separate experiment, wild-type mice were immunized i.m. with 10 μg of OVA protein plus SAS twice, with a two-week interval. One week after the last immunization, mononuclear splenocytes were generated by mincing whole mouse spleens, followed by the lysis of red blood cells and 70-μm filtration. Splenocytes were cultured in RPMI 1640 medium plus 10% heat-inactivated FBS, 100 U/mL penicillin, and 100 mg/mL streptomycin (Gibco, Dublin, Ireland).

### 2.4. Interferon-γ Enzyme-Linked Immunospot (ELISPOT) Assay

Antigen-specific IFN-γ-producing spot forming units (SFUs) were evaluated using mouse IFN-γ ELISPOT kits (BD Biosciences). Splenocytes were plated at 1 × 10^6^ cells/well onto purified IFN-γ antibody-coated PVDF-backed plates and stimulated with an RBD peptide pool (2 μg/mL each), 5 μg/mL individual peptide, or 5 μg/mL OVA_257–264_ at 37 °C for 2 days. In a separate experiment, splenocytes were stimulated with 10-mer peptides (5 μg/mL) in the presence of mouse IgG2a isotype as a control, anti-H2K^b^ and/or anti-H2D^b^ mAbs (20 μg/mL) at 37 °C for 2 days. The IFN-γ-positive SFUs were enumerated using an ELISPOT plate reader (Cellular Technology Ltd., Cleveland, OH, USA).

### 2.5. Flow Cytometry Intracellular Cytokine Staining

Splenocytes at 1 × 10^6^ cells/well were exposed to an RBD peptide pool (2 μg/mL for each peptide) or 5 μg/mL of each peptide in the presence of a protein transport inhibitor containing brefeldin A (BD Biosciences) for 12 h at 37 °C. Intracellular cytokine staining was performed according to a protocol modified from a previous paper [8]. The treated cells were incubated with LIVE/DEAD Fixable Red Dead Cell stain (Invitrogen) for 20 min at room temperature. The cells were washed, incubated with an anti-CD16/32 antibody (2.4G2) solution to block the Fc receptor, and stained for surface markers (CD3ε, CD4, and CD8 molecules), followed by intracellular IFN-γ cytokine staining using the Foxp3/transcription factor buffer set (eBioscience). The antibodies used were anti-CD3ε BUV737 (17A2), anti-CD4 APC (RM4–5), anti-CD8α PE-Cy7 (53–6.7), and anti-IFN-γ BV650 (XMG1.2). All the antibodies were obtained from BD Biosciences. The stained cells were acquired on a FACS Aria Fusion (BD Biosciences) and were analyzed via FlowJo^TM^ V10 (TreeStar, Ashland, OR, USA).

### 2.6. Statistical Analysis

All the analyses were performed using the PRISM software 9.0.0 (GraphPad, San Diego, CA, USA), and *p* values less than 0.05 (*p* < 0.05) were considered to be statistically significant.

## 3. Results

### 3.1. SARS-CoV-2 S_391–405_ Peptide Facilitates IFN-γ Production of CD8 T Cells in Splenocytes from Adjuvanted SARS-CoV-2 Spike RBD-Immunized Mice

**Figure 1:**
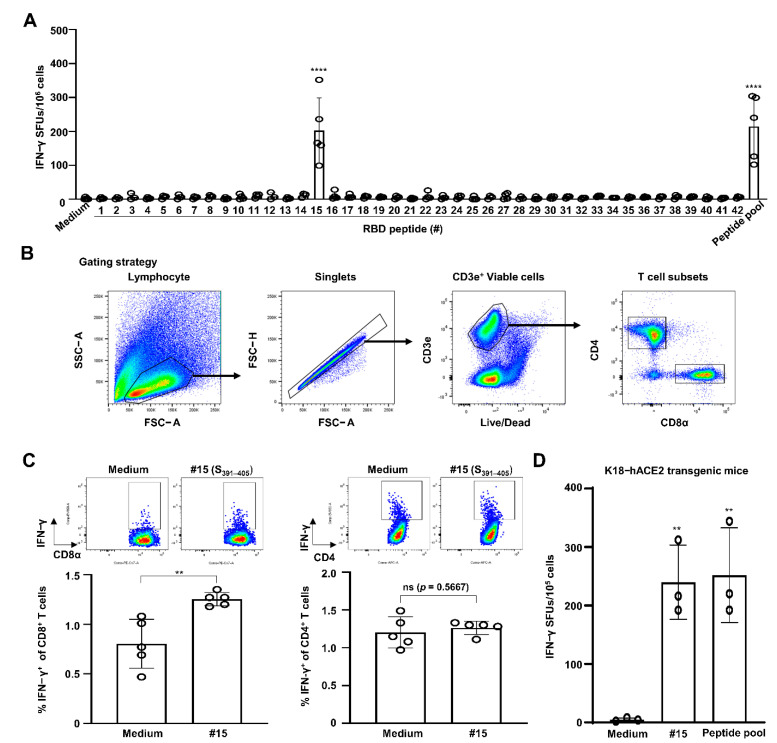
SARS-CoV-2 S_391–405_ elicits IFN-γ production by CD8 T cells in splenocytes of SARS-CoV-2 S-RBD protein-immunized C57BL/6 mice. C57BL/6 mice (*n* = 5) were immunized i.m. with RBD recombinant protein plus Sigma Adjuvant System (SAS) twice with a two-week interval. One week from the final immunization, cells were harvested from spleens of the immunized mice. (**A**) Splenocytes were simulated with 5 μg/mL of each RBD peptide or a peptide pool (each 2 μg/mL) for 2 days to evaluate CD8 CTL activity via an IFN-γ ELISPOT assay. Statistical significance was analyzed using one-way ANOVA with Tukey’s multiple comparison test (F_(43, 129)_ = 12.22, *p* < 0.0001) (**** *p* < 0.0001, medium vs. #15 or peptide pool). (**B**) Gating strategy for analysis of T cell subsets in flow cytometry. (**C**) Splenocytes stimulated with peptide #15 (corresponding to S_391–405_) at 5 μg/mL in the presence of brefeldin A for 12 h. Percentages of IFN-γ^+^ T cell subsets were analyzed by flow cytometry. Statistical significance was analyzed using two-sided unpaired *t*-tests (IFN-γ^+^ CD8 T cells: t_(8)_ = 3.928, ** *p* = 0.0044; IFN-γ^+^ CD4 T cells: t_(8)_ = 0.5976, *p* = 0.5667). (**D**) K18-hACE2 transgenic mice (*n* = 3) were immunized with SAS-adjuvanted RBD protein, and an IFN-γ ELISPOT assay was performed by stimulating splenocytes with peptide #15 or an RBD peptide pool. Statistical significance was analyzed using one-way ANOVA with Tukey’s multiple comparison test (F_(2, 6)_ = 16.54, *p* < 0.0036) (** *p* = 0.0068, medium vs. #15; ** *p* = 0.0053, medium vs. peptide pool). These experiments were performed three times, producing similar results. The bar graphs indicate the means with SDs. ns, not significant. Sharp-mark (#) indicates the order of individual peptide listed in overlapping 42 peptides.

To identify the CTL epitope(s) of the SARS-CoV-2 RBD, C57BL/6 mice (*n* = 5) were i.m. immunized twice with SARS-CoV-2 RBD recombinant protein along with SAS—composed of monophosphoryl lipid A and squalene—with a two-week interval. One week after the last immunization, an IFN-γ ELISPOT assay was performed by stimulating the splenocytes obtained from immunized mice with an RBD peptide pool of 42 synthetic RBD peptides with 15-mers overlapping by 5 aa (Table 1). As shown in Figure 1A, the number of IFN-γ SFUs was significantly increased in cells treated with 15th peptide out of 42 peptides (CFTNVYADSFVIRGD, corresponding to S_391–405_) (202 ± 96 SFUs) compared to that in the medium control (2 ± 3 SFUs) (*p* < 0.0001). The level of increase provoked by treatment with the S_391–405_ peptide was similar to the SFU values achieved via stimulation with the RBD peptide pool (214 ± 95 SFUs) (*p* < 0.0001). However, none of the other 41 individual RBD peptides induced the production of IFN-γ SFUs.

To confirm that S_391–405_-induced IFN-γ production was dependent on CD8 T cells but not CD4 T cells, splenocytes were subjected to intracellular IFN-γ staining. The gating strategy for flow cytometry employed is depicted in Figure 1B. In line with our expectations, the percentages of IFN-γ^+^ CD8 T cells were almost 1.7-fold higher in the S_391–405_-treated cells (1.25 ± 0.07%) than the medium-treated cells (0.80 ± 0.25%) (*p* = 0.0044) (Figure 1C). However, the percentages of IFN-γ^+^ CD4 T cells were similar between the S_391–405_- and medium-treated cells (*p* = 0.5667). Next, we assessed the SARS-CoV-2 RBD-specific CTL activity using K18-hACE2 transgenic mice (C57BL/6 background), which have been reported as an animal model suitable for SARS-CoV-2 studies [14]. When stimulating splenocytes from the RBD protein-immunized K18-hACE2 transgenic mice (*n* = 3) with the RBD peptide pool, the IFN-γ SFU numbers were also observed to be higher in the S_391–405_ peptide-stimulated cells (240 ± 64 SFUs) than in the control cells (5 ± 3 SFUs) (*p* = 0.0068). The extent of this finding is similar to the effect of stimulation with the RBD peptide pool (252 ± 81 SFUs) (*p* = 0.0053) (Figure 1D). These results suggest that the SARS-CoV-2 S_391–405_ peptide contains CTL epitope(s) in the C57BL/6 mouse model.

### 3.2. SARS-CoV-2 S_395–404_ Acts as the MHC Class I H-2K^b^/D^b^-Restricted Minimal CTL Epitope of SARS-CoV-2 Spike RBD

**Figure 2:**
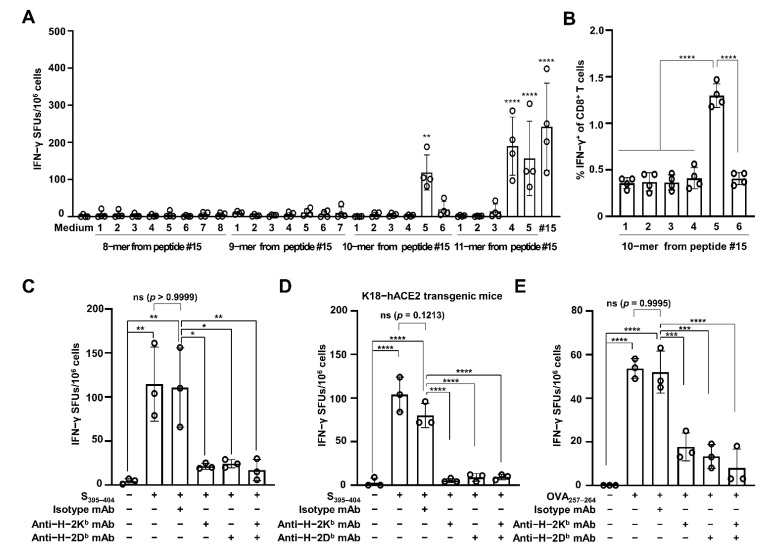
SARS-CoV-2 S_395–__404_ increases H-2K^b^/D^b^-restricted IFN-γ production. (**A**) Splenocytes were harvested from SAS-adjuvanted SARS-CoV-2 RBD protein-immunized C57BL/6 mice (*n* = 4) and stimulated with 8–11-mer peptides within the SARS-CoV-2 S_391–405_ region. CD8 CTL activity was evaluated by enumerating IFN-γ SFUs via the ELISPOT assay. Statistical significance was analyzed using one-way ANOVA with Tukey’s multiple comparison tests (F_(27, 84)_ = 13.37, *p* < 0.0001) (** *p* = 0.0023, medium vs. #5 of 10-mers; **** *p* < 0.0001, medium vs. #4 of 11-mers, #5 of 11-mers, medium vs. #15, or #15). (**B**) Percentages of IFN-γ-producing CD8 T cells were analyzed using 10-mer peptide-treated splenocytes via flow cytometry. Statistical significance was analyzed using one-way ANOVA with Tukey’s multiple comparison tests (F_(5, 18)_ = 63.64, *p* < 0.0001) (**** *p* < 0.0001). Splenocytes were harvested from adjuvanted SARS-CoV-2 RBD protein-immunized C57BL/6 mice (*n* = 3) (**C**) and K18-hACE2 transgenic mice (*n* = 3) (**D**) and then stimulated with the SARS-CoV-2 S_395–404_ peptide in the presence of anti-H-2K^b^ and/or anti-H-2D^b^ mAb for 2 days to measure IFN-γ SFUs via the ELISPOT assay. Statistical significance was analyzed using one-way ANOVA with Tukey’s multiple comparison tests (F_(5, 12)_ = 11.32, *p* = 0.003; F_(5, 12)_ = 55.3, *p* = 0.0001). *p* values are as follows: panel (**C**) ** *p* = 0.0022 between non-treatment and S_391–405_, ** *p* = 0.0029 between non-treatment and S_391–405_ plus isotype mA, * *p* = 0.0112 between S_391–405_ plus isotype mAb and S_391–405_ plus anti-H2-K^b^ mAb, * *p* = 0.0142 between S_391–405_ plus isotype mAb and S_391–405_ plus anti-H2-D^b^ mAb, ** *p* = 0.0079 between S_391–405_ plus isotype mAb and S_391–405_ plus anti-H2-K^b^/ D^b^ mAbs; (**D**) **** *p* < 0.0001 and *p* = 0.1213. (**E**) C57BL/6 mice (*n* = 3) were immunized i.m. with 10 μg of OVA protein plus SAS twice with a two-week interval. One week from the last immunization, splenocytes were harvested from the immunized mice and simulated with OVA_257–264_ peptide in the presence of anti-H-2K^b^ and/or anti-H-2D^b^ mAb. Two days after incubation, IFN-γ SFUs were enumerated via the ELISPOT assay. Statistical significance was analyzed using one-way ANOVA with Tukey’s multiple comparison tests (F_(5, 12)_ = 33.69, *p* < 0.0001) (**** *p* < 0.0001, *** *p* = 0.0002 between OVA_257–264_ plus isotype mAb and OVA_257–264_ plus anti-H2-K^b^ mAb, *** *p* = 0.0004 between OVA_257–264_ plus isotype mAb and OVA_257–264_ plus anti-H2-D^b^ mAb, and *p* = 0.9995 between OVA_257–264_ and OVA_257–264_ plus isotype mAb). These experiments were performed three times, with similar results. The bar graphs indicate the means with SDs. ns, not significant. Sharp-mark (#) indicates the order of individual peptide listed in overlapping 42 peptides.

As CD8 CTLs recognize foreign antigen peptides of 8–11 aa residues, we attempted to identify the minimum length of CTL epitope that could be recognized by major histocompatibility complex (MHC) class I on CD8 T cells. Splenocytes obtained from the RBD-immunized mice (*n* = 4) were harvested and subjected to IFN-γ ELISPOT with 8–11-mer peptides derived from SARS-CoV-2 S_391–405_ (Table 2). As shown in Figure 2A, none of the 8- or 9-mer peptides triggered a significant generation of IFN-γ SFUs. Of the 10-mer peptides, however, a significantly higher number of IFN-γ SFUs was observed after treatment with peptide #5 only (VYADSFVIRG, corresponding to S_395–404_; 119 ± 47 SFUs) than with the medium control (2 ± 2 SFUs) (*p* = 0.0023). Two 11-mer peptides, including S_395–404_, also significantly augmented IFN-γ SFU numbers (190 ± 78 SFUs for peptide #4, corresponding to S_394–404_; 157 ± 100 SFUs for peptide #5, corresponding to S_395–405_) (*p* < 0.0001). Flow cytometry also showed that the percentages of IFN-γ^+^ CD8 T cells were significantly higher (three-fold) in the cells treated with only the S_395–404_ peptide (1.3 ± 0.12%) than in those treated with other 10-mer peptides (0.36 ± 0.06, 0.37 ± 0.1, 0.37 ± 0.08, 0.41 ± 0.11, and 0.41 ± 0.07% for peptide numbers 1, 2, 3, 4, and 6, respectively) (*p* < 0.0001) (Figure 2B). These results indicate that the SARS-CoV-2 S_395–404_ peptide is a minimal epitope for CTLs specific for the SARS-CoV-2 spike protein RBD.

Finally, we tested MHC restriction for the recognition of the SARS-CoV-2 S_395–404_ peptide using antibodies blocking MHC class I, such as anti-H-2K^b^ and anti-H-2D^b^ mAbs, via an IFN-γ ELISPOT assay. The S_395–404_-increasing IFN-γ SFUs (115 ± 42 SFUs) were similar to those for cells treated with the mAb isotype (111 ± 45 SFUs) (*p* = 0.9999) (Figure 2C). However, the IFN-γ SFUs were significantly blocked by treatment with anti-H-2K^b^ mAb (21 ± 3 SFUs; *p* = 0.0112), anti-H-2D^b^ mAb (24 ± 5 SFUs; *p* = 0.0142), or a mixture of both (17 ± 12 SFUs; *p* = 0.0079), as compared with treatment with the mAb isotype. Furthermore, when using splenocytes from the SARS-CoV-2 spike RBD-immunized K18-hACE2 mice (*n* = 3), the S_395–404_ peptide significantly augmented IFN-γ SFU numbers (104 ± 20 SFUs) compared to the medium control (3 ± 5 SFUs) (*p* < 0.0001) (Figure 2D). Similar S_395–404_-inducing IFN-γ SFU numbers were observed in cells treated with the control mAb isotype (80 ± 14 SFUs) (*p* = 0.1213). However, these numbers were significantly decreased by the addition of anti-H-2K^b^ mAb (5 ± 3 SFUs), anti-H-2D^b^ mAb (9 ± 4 SFUs), or a mixture of both (9 ± 3 SFUs) (*p* < 0.0001). To confirm our results, we tested splenocytes from OVA-immunized mice (*n* = 3) using the well-known CTL epitope OVA_257–264_ [16]. After the stimulation of splenocytes with OVA_257–264_, significant production of IFN-γ SFUs was observed in the OVA-immunized mice (54 ± 5 SFUs and 52 ± 10 SFUs, in the absence and presence of isotype mAb, respectively), as compared with that in the medium control (0 SFU) (*p* < 0.0001). However, treatment with both anti-H-2K^b^ and anti-H-2D^b^ mAbs significantly inhibited OVA_257–264_-stimulated IFN-γ SFU production (18 ± 6 SFUs after treatment with anti-H-2K^b^ mAb, *p* = 0.0002; 13 ± 6 SFUs after treatment with anti-H-2D^b^ mAb, *p* = 0.0004; 8 ± 9 SFUs after treatment with anti-H-2K^b^/D^b^ mAbs, *p* = 0.0001) (Figure 2E). Collectively, these data indicate that SARS-CoV-2 S_395–404_ is the MHC class I H-2K^b^/D^b^-restricted CTL epitope of the SARS-CoV-2 spike protein RBD in the C57BL/6 mouse model.

## 4. Discussion

The SARS-CoV-2 S_395–404_ peptide, VYADSFVIRG, is localized upstream of the receptor-binding motif within the RBD region and is not absolutely identical to the SARS-CoV S_382–391_ peptide (VYADSFVVKG, Accession no. YP_009825051.1). A previous study reported SARS-CoV S_436–443_ (YNKKYRYL) and S_525–532_ (VNFNFNGL) as H-2K^b^-restricted epitopes [17]; however, these sequences were not found in the SARS-CoV-2 S protein. The SARS-CoV-2 S_526–533_ peptide (GPKKSTNL) was recently reported to be the H-2K^d^-restricted CTL epitope in BALB/c mice [11]. However, the S_526–533_-containing peptide #42 did not induce generation of IFN-γ^+^ cells from immunized C57BL/6 mice, as shown in Figure 1A, suggesting that SARS-CoV-2 S_395–404_ might be a unique CTL epitope of the SARS-CoV-2 S RBD in the C57BL/6 mouse model. Similarly to our results, some peptides, including glycoprotein_33–43_ of lymphocytic choriomeningitis virus [18] and the ovalbumin peptide (OVA_257–264_) [16], have been reported to be the H-2K^b^/D^b^-binding epitope. Further studies are needed to measure the binding affinity of SARS-CoV-2 S_395–404_ peptide to MHC class I molecules.

Various domains within SARS-CoV S protein are considered SARS-CoV-2 vaccine targets. RBD domain is one of the important antigens for SARS-CoV-2 vaccines and several RBD-based vaccine candidates have been tested in clinical trials [19]. Of them, lipid nanoparticle-formulated RBD mRNA vaccine candidates are reported to induce both neutralizing antibody response and T cell activation in preclinical [20,21] and clinical fields [22]. Aluminum-adjuvanted RBD vaccine candidate is reported to increase neutralizing antibody response rather than T cell immunity in mice, rabbits and non-human primates [23]. A recombinant subunit vaccine candidate containing the RBD of SARS-CoV-2 and the Fc fragment of human lgG is shown that increase neutralizing antibody response only in a mouse model [24]. Several studies have reported that SARS-CoV-specific CD8 CTLs substantially protected against SARS-CoV infection in mice [25] and were increased in mice that were vaccinated with SARS-CoV S DNA as the vaccine antigen [26]. As an increase in CTL activity specific to SARS-CoV-2 has been reported in the peripheral blood mononuclear cells from COVID-19 convalescent patients [27], SARS-CoV-2-specific CTLs may have a protective role in SARS-CoV-2 infection. Translation of the vaccine immunity in human subjects may be difficult to accurately predict from animal models, but animal models is most useful in stratifying the vaccine candidates through careful evaluation of vaccine efficacy before clinical trials [28]. Many experimental approaches aimed at quantifying SARS-CoV-2-specific CTL activity have used RBD peptide pools having limitations such as expensive and time-consuming synthesis of peptides [8,10].

Our findings would be useful for resolving these problems and could be beneficial for evaluating SARS-CoV-2 spike RBD-specific CTL responses and studying T cell functions. Moreover, these findings may be helpful for the generation of a tetrameric MHC–peptide complex that can be used to directly quantify and visualize SARS-CoV-2 S RBD-specific T cells [29]. Taken together, the identification of SARS-CoV-2 S_395–404_ as the CTL epitope may facilitate the evaluation of CTL activity of RBD-based COVID-19 vaccine candidates in a mouse model and the investigation of CTL function in SARS-CoV-2 infection.

## 5. Conclusions

Identification of the SARS-CoV-2 spike RBD-specific CTL epitope is necessary to exactly analyze SARS-CoV-2-specific CTL activity and to solve problems of costly and time-consuming peptide synthesis for peptide pools. Our finding of SARS-CoV-2 S_395–404_ as the CTL epitope is helpful in the study of SARS-CoV-2-specific CTL functions and the evaluation of CTL activity of RBD-based COVID-19 vaccine candidates using a mouse model.

## Figures and Tables

**Table 1:** List of tested 15-mer receptor-binding domain (RBD) peptides of SARS-CoV-2 spike region.

Peptide ID	Start	End	Sequence
1	321	335	QPTESIVRFPNITNL
2	326	340	IVRFPNITNLCPFGE
3	331	345	NITNLCPFGEVFNAT
4	336	350	CPFGEVFNATRFASV
5	341	355	VFNATRFASVYAWNR
6	346	360	RFASVYAWNRKRISN
7	351	365	YAWNRKRISNCVADY
8	356	370	KRISNCVADYSVLYN
9	361	375	CVADYSVLYNSASFS
10	366	380	SVLYNSASFSTFKCY
11	371	385	SASFSTFKCYGVSPT
12	376	390	TFKCYGVSPTKLNDL
13	381	395	GVSPTKLNDLCFTNV
14	386	400	KLNDLCFTNVYADSF
15	391	405	**CFTNVYADSFVIRGD**
16	396	410	YADSFVIRGDEVRQI
17	401	415	VIRGDEVRQIAPGQT
18	406	420	EVRQIAPGQTGKIAD
19	411	425	APGQTGKIADYNYKL
20	416	430	TKIADYNYKLPDDFT
21	421	435	YNYKLPDDFTGCVIA
22	426	440	PDDFTGCVIAWNSNN
23	431	445	GCVIAWNSNNLDSKV
24	436	450	WNSNNLDSKVGGNYN
25	441	455	LDSKVGGNYNYLYRL
26	446	460	GGNYNYLYRLFRKSN
27	451	465	YLYRLFRKSNLKPFE
28	456	470	FRKSNLKPFERDIST
29	461	475	LKPFERDISTEIYQA
30	466	480	RDISTEIYQAGSTPC
31	471	485	EIYQAGSTPCNGVEG
32	476	490	GSTPCNGVEGFNCYF
33	481	495	NGVEGFNCYFPLQSY
34	486	500	FNCYFPLQSYGFQPT
35	491	505	PLQSYGFQPTNGVGY
36	496	510	GFQPTNGVGYQPYRV
37	501	515	NGVGYQPYRVVVLSF
38	506	520	QPYRVVVLSFELLHA
39	511	525	VVLSFELLHAPATVC
40	516	530	ELLHAPATVCGPKKS
41	521	535	PATVCGPKKSTNLVK
42	526	540	CGKKSTNLVKNKCVN

The peptide capable of enhancing cytotoxic T lymphocyte (CTL) activity is highlighted in bold.

**Table 2:** List of tested 8–11-mer peptides within SARS-CoV-2 spike RBD_391–405_ region.

Peptide ID	Start	End	Sequence
15-8-1	391	398	CFTNVYAD
15-8-2	392	399	FTNVYADS
15-8-3	393	400	TNVYADSF
15-8-4	394	401	NVYADSFV
15-8-5	395	402	VYADSFVI
15-8-6	396	403	YADSFVIR
15-8-7	397	404	ADSFVIRG
15-8-8	398	405	DSFVIRGD
15-9-1	391	399	CFTNVYADS
15-9-2	392	400	FTNVYADSF
15-9-3	393	401	TNVYADSFV
15-9-4	394	402	NVYADSFVI
15-9-5	395	403	VYADSFVIR
15-9-6	396	404	YADSFVIRG
15-9-7	397	405	ADSFVIRGD
15-10-1	391	400	CFTNVYADSF
15-10-2	392	401	FTNVYADSFV
15-10-3	393	402	TNVYADSFVI
15-10-4	394	403	NVYADSFVIR
15-10-5	395	404	**VYADSFVIRG**
15-10-6	396	405	YADSFVIRGD
15-11-1	391	401	CFTNVYADSFV
15-11-2	392	402	FTNVYADSFVI
15-11-3	393	403	TNVYADSFVIR
15-11-4	394	404	**NVYADSFVIRG**
15-11-5	395	405	**VYADSFVIRGD**

The peptide capable of enhancing cytotoxic T lymphocyte (CTL) activity is highlighted in bold.

## Data Availability

The data presented in this study are available on request from the corresponding author.

## References

[1] Cohen J., Normile D. (2020). New SARS-like virus in China triggers alarm. Science.

[2] World Health Organization Coronavirus Disease 2019 (COVID-19) Situation Report—62. https://www.who.int/docs/default-source/coronaviruse/situation-reports/20200322-sitrep-62-covid-19.pdf?sfvrsn=f7764c46_2.

[3] Mullard A. (2020). COVID-19 vaccine development pipeline gears up. Lancet.

[4] Graham B.S. (2020). Rapid COVID-19 vaccine development. Science.

[5] Lan J., Ge J., Yu J., Shan S., Zhou H., Fan S., Zhang Q., Shi X., Wang Q., Zhang L. (2020). Structure of the SARS-CoV-2 spike receptor-binding domain bound to the ACE2 receptor. Nature.

[6] Appay V., Douek D.C., Price D.A. (2008). CD8+ T cell efficacy in vaccination and disease. Nat. Med..

[7] Wang Z., Yang X., Zhou Y., Sun J., Liu X., Zhang J., Mei X., Zhong J., Zhao J., Ran P. (2020). COVID-19 Severity Correlates with Weaker T-Cell Immunity, Hypercytokinemia, and Lung Epithelium Injury. Am. J. Respir. Crit. Care Med..

[8] Corbett K.S., Edwards D.K., Leist S.R., Abiona O.M., Boyoglu-Barnum S., Gillespie R.A., Himansu S., Schafer A., Ziwawo C.T., DiPiazza A.T. (2020). SARS-CoV-2 mRNA vaccine design enabled by prototype pathogen preparedness. Nature.

[9] Smith T.R.F., Patel A., Ramos S., Elwood D., Zhu X., Yan J., Gary E.N., Walker S.N., Schultheis K., Purwar M. (2020). Immunogenicity of a DNA vaccine candidate for COVID-19. Nat. Commun..

[10] van Doremalen N., Lambe T., Spencer A., Belij-Rammerstorfer S., Purushotham J.N., Port J.R., Avanzato V.A., Bushmaker T., Flaxman A., Ulaszewska M. (2020). ChAdOx1 nCoV-19 vaccine prevents SARS-CoV-2 pneumonia in rhesus macaques. Nature.

[11] Muraoka D., Situo D., Sawada S.I., Akiyoshi K., Harada N., Ikeda H. (2020). Identification of a dominant CD8(+) CTL epitope in the SARS-associated coronavirus 2 spike protein. Vaccine.

[12] Golden J.W., Cline C.R., Zeng X., Garrison A.R., Carey B.D., Mucker E.M., White L.E., Shamblin J.D., Brocato R.L., Liu J. (2020). Human angiotensin-converting enzyme 2 transgenic mice infected with SARS-CoV-2 develop severe and fatal respiratory disease. JCI Insight.

[13] Johansen M.D., Irving A., Montagutelli X., Tate M.D., Rudloff I., Nold M.F., Hansbro N.G., Kim R.Y., Donovan C., Liu G. (2020). Animal and translational models of SARS-CoV-2 infection and COVID-19. Mucosal Immunol..

[14] Oladunni F.S., Park J.G., Pino P.A., Gonzalez O., Akhter A., Allue-Guardia A., Olmo-Fontanez A., Gautam S., Garcia-Vilanova A., Ye C. (2020). Lethality of SARS-CoV-2 infection in K18 human angiotensin-converting enzyme 2 transgenic mice. Nat. Commun..

[15] Winkler E.S., Bailey A.L., Kafai N.M., Nair S., McCune B.T., Yu J., Fox J.M., Chen R.E., Earnest J.T., Keeler S.P. (2020). SARS-CoV-2 infection in the lungs of human ACE2 transgenic mice causes severe inflammation, immune cell infiltration, and compromised respiratory function. bioRxiv.

[16] Ip P.P., Nijman H.W., Daemen T. (2015). Epitope Prediction Assays Combined with Validation Assays Strongly Narrows down Putative Cytotoxic T Lymphocyte Epitopes. Vaccines.

[17] Zhou M., Xu D., Li X., Li H., Shan M., Tang J., Wang M., Wang F.S., Zhu X., Tao H. (2006). Screening and identification of severe acute respiratory syndrome-associated coronavirus-specific CTL epitopes. J. Immunol..

[18] Hudrisier D., Oldstone M.B., Gairin J.E. (1997). The signal sequence of lymphocytic choriomeningitis virus contains an immunodominant cytotoxic T cell epitope that is restricted by both H-2D(b) and H-2K(b) molecules. Virology.

[19] Min L., Sun Q. (2021). Antibodies and Vaccines Target RBD of SARS-CoV-2. Front. Mol. Biosci..

[20] Zhang N.N., Li X.F., Deng Y.Q., Zhao H., Huang Y.J., Yang G., Huang W.J., Gao P., Zhou C., Zhang R.R. (2020). A Thermostable mRNA Vaccine against COVID-19. Cell.

[21] Vogel A.B., Kanevsky I., Che Y., Swanson K.A., Muik A., Vormehr M., Kranz L.M., Walzer K.C., Hein S., Guler A. (2021). BNT162b vaccines protect rhesus macaques from SARS-CoV-2. Nature.

[22] Sahin U., Muik A., Derhovanessian E., Vogler I., Kranz L.M., Vormehr M., Baum A., Pascal K., Quandt J., Maurus D. (2020). COVID-19 vaccine BNT162b1 elicits human antibody and TH1 T cell responses. Nature.

[23] Yang J., Wang W., Chen Z., Lu S., Yang F., Bi Z., Bao L., Mo F., Li X., Huang Y. (2020). A vaccine targeting the RBD of the S protein of SARS-CoV-2 induces protective immunity. Nature.

[24] Liu Z., Xu W., Xia S., Gu C., Wang X., Wang Q., Zhou J., Wu Y., Cai X., Qu D. (2020). RBD-Fc-based COVID-19 vaccine candidate induces highly potent SARS-CoV-2 neutralizing antibody response. Signal Transduct. Target Ther..

[25] Channappanavar R., Fett C., Zhao J., Meyerholz D.K., Perlman S. (2014). Virus-specific memory CD8 T cells provide substantial protection from lethal severe acute respiratory syndrome coronavirus infection. J. Virol..

[26] Zhao K., Yang B., Xu Y., Wu C. (2010). CD8+ T cell response in HLA-A*0201 transgenic mice is elicited by epitopes from SARS-CoV S protein. Vaccine.

[27] Habel J.R., Nguyen T.H.O., van de Sandt C.E., Juno J.A., Chaurasia P., Wragg K., Koutsakos M., Hensen L., Jia X., Chua B. (2020). Suboptimal SARS-CoV-2-specific CD8(+) T cell response associated with the prominent HLA-A*02:01 phenotype. Proc. Natl. Acad. Sci. USA.

[28] Golding H., Khurana S., Zaitseva M. (2018). What Is the Predictive Value of Animal Models for Vaccine Efficacy in Humans? The Importance of Bridging Studies and Species-Independent Correlates of Protection. Cold Spring Harb. Perspect. Biol..

[29] Sims S., Willberg C., Klenerman P. (2010). MHC-peptide tetramers for the analysis of antigen-specific T cells. Expert Rev. Vaccines.

